# 

**Published:** 1896-11-28

**Authors:** 


"The standard of highest purity at present attainable."—*Lancet*.

CADBURY'S COCOA

NO ALKALIES USED.

### THE HOSPITAL

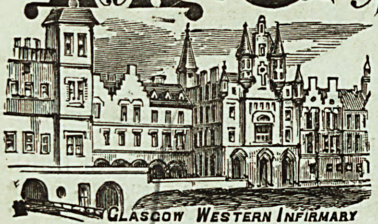

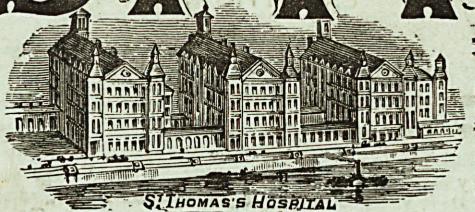

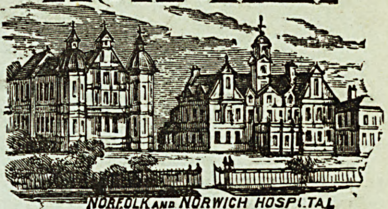

No. 531.—Vol. XXI.

SATURDAY, NOV. 28TH, 1896.

WITH EXTRA NURSING SUPPLEMENT.
[Registered at the General Post Office as a Newspaper for transmission in the United Kingdom and Abroad.]

PRICE TWOPENCE.

Per Annum, 10s. 6d., post free.

Monthly Parts, 6d.; post free, 8d.

Ditto per Annum, 8s. 6d., post free.

